# Laparoscopic Surgery in the treatment of children with Choledochal Cyst

**DOI:** 10.12669/pjms.35.3.85

**Published:** 2019

**Authors:** Xiushui Qu, Lihua Cui, Junchao Xu

**Affiliations:** 1 *Xiushui Qu, Department of Pediatric Surgery, Binzhou People’s Hospital, Shandong, 256610, China*; 2 *Lihua Cui, Department of Hand and Foot Surgery, Binzhou People’s Hospital, Shandong, 256610, China*; 3 *Junchao Xu, Department of Pediatric Surgery, Binzhou People’s Hospital, Shandong, 256610, China*

**Keywords:** Laparoscopic surgery, Laparotomy, Choledochal cyst, Children

## Abstract

**Objective:**

To evaluate the clinical effectiveness of laparoscopic surgery in the treatment of children with choledochal cyst.

**Methods:**

Seventy-six children with congenital choledochal cyst who were admitted to our hospital between February 2016 and April 2017 were selected as research subjects. They were evenly divided into an observation group and a control group using random number table, 38 each group. Patients in the observation group underwent laparoscopic surgery, while patients in the control group underwent the traditional laparotomy. Surgery related indicators and prognosis were compared between the two groups.

**Results:**

The incision size and intraoperative bleeding volume of the observation group were significantly smaller than those of the control group (P<0.05). The time of passage of flatus and time to take food of the observation group were easier than those of the control group, and the duration of hospitalization and parenteral nutrition of the former was significantly shorter than those of the latter, and the difference had statistical significance (P<0.05). The incidence of postoperative complications in the observation group was 2.6%, significantly lower than that in the control group (10.5%) (P<0.05). There was no recurrence in the observation group during the follow-up period, but there were 5 cases of recurrence (13.1%) in the control group; the difference was statistically significant (P<0.05).

**Conclusion:**

Compared with the traditional laparotomy, laparoscopic surgery conforms more to the concept of modern medical minimally invasive treatment and has a significant clinical effect in the treatment of congenital choledochal cyst in children. It can effectively promote the disappearance of clinical symptoms and signs, reduce the incidence of postoperative complications and disease recurrence, and improve the surgical efficacy, suggesting high clinical significance and application values.

## INTRODUCTION

Pediatric choledochal cyst, also known as biliary dilatation, is a common clinical biliary malformation disease and congenital abnormality in children.^1^,^2^ In recent years, the incidence of pediatric choledochal cyst is on the rise. The incidence of the disease in Asian countries is much higher than that in Europe and America.^3^ The main clinical symptoms of pediatric choledochal cyst include intermittent upper abdominal pain, mass and jaundice in right upper abdomen, nausea, vomiting and anorexia.^4^ It should be treated by surgery immediately after diagnosis. If the treatment is delayed, severe complications including pancreatitis, pyogeniccholangitis, rupture and perforation of cyst and liver cirrhosis induced by repeated biliary tract infection and obstruction of biliary tract may appear, which can threaten lives of children.^5^

Cystectomy and Roux-en-Y hepatojejunostomy are the standard surgeries for the treatment of choledochal cyst currently. The traditional laparotomy can cause large surgical trauma, large amount of bleeding and high risks of postoperative complications such as intestinal adhesion, which may bring great pains to children, and moreover the scar is obvious and the surgical efficacy is not satisfactory.^6^ Laparoscopic choledochal cystectomy was proposed by Farello et al.^7^ in 1995. It has advantages of small surgical trauma, short hospitalization time and fast postoperative recovery. Roux-en-Y hepatojejunostomy is one of surgeries with high difficulty in clinical pediatric surgery. Compared to the traditional laparotomy, laparoscopic surgery can expose surgical field better to further display the relationship between cyst and surrounding tissues, which is beneficial to the accurate operation of doctors.^8^-^10^ However, laparoscopic choledochal cystectomy and Roux-en-Y hepatojejunostomy requires excellent skills of operators, and the operation experience needs to be accumulated through clinical practices. Diao et al. found that the surgical time shortened when the number of cases that the operator treated exceeded 35.^11^ The safety and effectiveness of laparoscopic surgery remains to be the focus. Such kind of prospective analysis and controlled study is seldom. In this study, the clinical difference of laparoscopic surgery and laparotomy in the treatment of pediatric choledochal cyst was analyzed to explore the advantages, disadvantages of laparoscope in the treatment of congenital choledochal cyst, which provides a reference and theoretical basis for the application of laparoscope in the treatment of pediatric choledochal cyst.

## METHODS

Seventy-six children with congenital choledochal cyst who were treated in the hospital between February 2016 and April 2017 were selected as research subjects. Children who conformed to diagnostic criteria of choledochal cyst, had different severity of clinical symptoms such as abdominal pain, fever and jaundice, had normal results of liver and gallbladder functions, and could undergo laparoscopy or radiography were included, but those who had disturbance of blood coagulation and dysfunction of platelet were excluded. The children were divided into an observation group and a control group using random number table, 38 each group. The family members of the children were informed with the content of the study and signed informed consent, and this study has been approved by the ethics committee of Binzhou People’s Hospital.

### Surgical Method

(1) Laparoscopic surgery group: Children in the observation group underwent choledochal cystectomy and Roux-en-Y hepatojejunostomy. General anesthesia was carried out firstly through endotracheal intubation. The children took a supine position, with the lower back supported. Three-port method was used. The operator stood at the right hand of the children, and the assistant stood at the left side of the children. Cholangiography was performed through puncture at the bottom of the cholecyst to understand the distribution of the bile duct and pancreiatic duct. The liver was suspended to expose the hepatic portal. The cholecyst was stripped through electrocoagulation. The cystic duct was identified. The anterior abdominal wall of cyst was incised and the cyst was dissociated towards the distal end. The anterior wall of the cyst was opened at the site near the duodenum. Then the cyst fluid was removed. The posterior wall of the cyst was transversed, and the portal vein and artery were paid due attention to. The distal end of the cyst was dissociated to the junction of pancreatic bile tracts using ultrasound knife and then ligatured. The duodenum and pancreiatic duct were also paid attention to. Then the proximal end of the cyst was dissociated. The left and right hepatic duct openings and the existence of accessory hepatic duct were determined according to intraoperative cholangiography. The cyst was completely resected, and the blood supply of the hepatic duct was protected. Under the assistance of laparoscope, transverse colon was pulled towards the direction of head to identify the position of Traitz ligament. Umbilical trocar was removed. Then 18.5 cm of jejunum was taken at the site which was 10 cm away from Traitz ligament and processed by Roux-Y anastomosis using 5-0 absorbable thread. The intestinal canal was sent back to the abdominal cavity. The jejunum was lifted and was anastomosed with the hepatic duct using 5-0 absorbable thread. The abdominal cavity was thoroughly washed. Drainage tube was indwelled. The trocar was removed if there was no bleeding in the abdominal cavity, and the incision was sutured. (2) Laparotomy group: The anesthesia and surgical procedures of the control group were nearly the same with the laparoscopic surgery group, but a transverse incision was made at the right upper abdomen.

After surgery, children in the two groups were given relevant treatment to resist infection, protect the liver and supplement fluid and nutrition.

### Observational Indicators

The intraoperative indicators including average operation duration, intraoperative bleeding volume and size of incision were compared between the two groups. The postoperative indicators including passage of gas by anus, time to take food, duration of hospitalization, duration of parenteral nutrition and complications were observed. The prognosis was compared between the two groups. The children were mainly followed up via phone after surgery, and some of them received outpatient follow-up. Some data were collected from some of the children were hospitalized again because of complication or other reasons. Children in the two groups were followed up for 3 to 12 months.

### Statistical processing

The obtained data were statistically analyzed using SPSS ver. 21.0. Measurement data were expressed by Mean±SD. Comparison between groups was performed using *t* test. Enumeration data were compared using Chi-square test. P<0.05 meant difference had statistical significance

## RESULTS

No significant difference was found between the two groups in age, gender and weight (P>0.05, Table-I).Compared to the control group, the observation group had smaller surgical incision and less intraoperative blood loss, and the differences were statistically significant (P<0.05). The duration of surgery of the observation group was longer than that of the control group, but the difference had no statistical significance (P>0.05, Table-II).

**Table-I:** General data between the two groups.

Groups	Age (year)	Gender		Length of	Diameter of	Type	

		Male	Female	cyst (cm)	cyst (cm)	Cyst	Fusiform
Observation group	4.52±3.26	10	28	5.91±3.17	4.38±2.47	30	8
Control group	3.56±3.14	12	26	6.97±2.87	5.17±3.25	28	10
t/X^2^	0.718	1.926	0.127	1.146	1.473		
P	>0.05	>0.05	>0.05	>0.05	>0.05		

**Table-II:** Intraoperative condition between the two groups.

Group	Duration of surgery (h)	Size of incision (mm)	Intraoperative blood loss (mL)
Observation group	4.06±0.47	1.60±0.41	8.81±4.12
Control group	3.19±0.45	8.17±1.46	19.04±13.06
t	1.715	14.867	5.927
P	>0.05	<0.05	<0.05

The time of passage of flatus and time to take food of the observation group were significantly shorter than those of the control group, and the duration of hospitalization and parenteral nutrition of the former was significantly shorter than that of the latter; the differences were not statistically significant (P<0.05). In the observation group, one case (5.3%) had temporary bile leakage, but cured after unobstructed drainage, which was considered being related to insufficient experience. In the control group, four children had postoperative complications (10.5%); two had intestinal obstruction, one had pneumonia and one had cholangitis. All of them cured after conservative treatment. There was a significant difference of incidence of complication between the two groups (P<0.05, (Table-III).

**Table-III:** Postoperative evaluation indicators between the two groups.

Group	Time of passage of flatus (d)	Time to take food (d)	Duration of hospitalization (d)	Duration of parenteral nutrition (d)	Incidence of postoperative complications (%)
Observation group	2.29±1.13	3.26±1.13	9.81±1.96	4.27±1.14	2.6
Control group	3.43±1.22	4.64±1.23	11.72±3.16	5.43±1.22	10.5
t/X^2^	5.058	6.172	3.878	5.059	2.571
P	<0.05	<0.05	<0.05	<0.05	<0.05

All the children with choledochal cyst were re-examined by blood routine examination, liver function examination and abdominal B-ultrasonography three days after operation. The long-term follow-up rate was 100% in both groups. None of the 76 children had anastomotic stenosis, cholangitis, pancreatitis and jaundice. The eating and defecation conditions of the children were good. Abdominal B-ultrasonography reexamination did not find liver abnormality, biliary tract dilatation and jaundice in those patients. There was no significant abnormality of liver function in the follow-up period. There was no recurrence in the observation group, but there were 5 cases of recurrence (13.1%) in the control group; there was no significant difference between the two groups (X^2^=4.133, P<0.05).

## DISCUSSION

Laparoscopic surgery tends to play a more important tole in the treatment of pediatric surgical disease because of its advantages of minimal invasion and wide field of vision, and the application of laparoscope in the treatment of choledochal cyst has been reported to have increased.^12^ As children have small volume of abdominal cavity, poor tolerance to pneumoperitoneum and precise biliary-intestinal anastomosis, laparoscopic choledochal cystectomy are challenging. Foreign scholars compared the short-term efficacy of laparoscope and open surgery in the treatment of choledochal cyst and found that laparoscopic surgery had the advantages of small trauma, fast recovery and short hospital stay and it had no influence on the incidence of postoperative complications,^13^,^14^ which was consistent with the research results of this study. Diao et al. in China found that laparoscopic surgery could significantly shorten the postoperative recovery time and the incidence of mid-term complications was lower than open surgery.^15^ We think that laparoscope has great advantages in the treatment of choledochal cyst. For example, the field of vision is wide under laparoscope and laparoscope can clearly display the appearance of cyst and its relationship with surrounding organs after 30° rotation. Moreover, laparoscope has the function of amplification. It can clearly display blood vessels on the surface of cyst walls, which is beneficial to preventive hemostasis. The intrahepatic bile duct and common bile duct can be probed to thoroughly clean protein plug and calculus through adjusting laparoscope.^16^ In laparoscopic surgery, the intestinal canal exposes for a short time and the interference to the abdominal cavity is small; therefore, the recovery of intestinal function is fast, and postoperative hospitalization time is shortened.^17^,^18^ Moreover the scar is not obvious as the incision is small, which is beneficial to the mental recovery of children.^19^

The results of the present study showed that the incision in the observation group was smaller than that in the control group, the amount of bleeding of the former was less than that of the latter, the observation group took food and exhausted by anus earlier than the control group. The duration of hospitalization and parenteral nutrition of the observation group were significantly shorter than that of the control group; the difference had statistical significance (P<0.05). These results have indicated that laparoscopic surgery was helpful to promote the disappearance of various clinical symptoms and signs in children, which is generally consistent with the research results of Akaraviputh et al.^20^ The results of this study also showed that there were no postoperative complications in the observation group and there were four cases (25.00%) of incision infection in the control group (P<0.05). It suggested that laparoscopic surgery could effectively reduce the incidence of postoperative complications, which might be related to less exposure during operation. There was no recurrence in the observation group and two cases (12.50%) in the control group during the follow-up period, but the difference had no statistical significance (P>0.05). It indicated that laparoscopic surgery was more effective in the treatment of children with congenital choledochal cyst and had ideal long-term efficacy.

Based on the author’s surgical experience and literature review, the key points of laparoscopic choledochal cystectomy and hepatojejunostomy techniques are summarized as follows. Intraoperative cholangiography is essential to fully understand the distribution and relationship of bile duct and pancreatic duct. Suspending the liver to fully expose the porta hepatis is conducive to dissociate cyst and biliointestinal anastomosis and avoids the use of liver support, which is more in line with the concept of minimally invasive. Dissociating cyst should be as close as possible to the wall of cyst. The anterior wall of cyst is opened near the duodenum, and cyst fluid should be removed, which is conducive to the resection of cyst. The treatment of proximal bile duct should be paid due attentions. After the position of the left and right hepatic duct openings are identified, bellmouth is trimmed. For fusiform type, the distal end of cyst should be carefully treated. Protein emboli existing in the common tube is washed away after catheterization to prevent postoperative pancreatitis. The distal common bile duct should be sutured to prevent postoperative pancreatic leakage. As to choledochojejunostomy, the blood supply of proximal hepatic duct should be protected. The opening needs to be trimmed neatly, and anterior wall and posterior wall are fully anastomosed. The needle spacing should be as small as possible to ensure anastomosis and avoid postoperative anastomotic stenosis.

## CONCLUSION

Laparoscopic surgery has the advantages of smaller trauma, fast recovery and good prognosis for children. With the development of laparoscopic instruments and the improvement of laparoscopic techniques, laparoscopic surgery is expected to become the standard operation for choledochal cyst. The size of samples in this study was small, and they were all retrospective data. Randomized controlled trials involving large sample size are needed to compare the traditional laparotomy and laparoscopic surgery.

### Authors’ Contribution

**XSQ:** Study design, data collection and analysis.

**XSQ & LHC:** Manuscript preparation, drafting and revising.

**XSQ & JCX:** Review and final approval of manuscript.
